# Changes in Metallothionein Level in Rat Hepatic Tissue after Administration of Natural Mouldy Wheat

**DOI:** 10.3390/ijms10031138

**Published:** 2009-03-12

**Authors:** Anna Vasatkova, Sarka Krizova, Vojtech Adam, Ladislav Zeman, Rene Kizek

**Affiliations:** 1 Department of Animal Nutrition and Forage Production, Faculty of Agronomy, Mendel University of Agriculture and Forestry, Zemedelska 1, CZ-613 00 Brno, Czech Republic; E-Mails: A.Vasatkova@seznam.cz (A.V.); xkrizov8@node.mendelu.cz (S.K.); ilabo@seznam.cz (V.A.); zeman@mendelu.cz (L.Z.); 2 Department of Chemistry and Biochemistry, Faculty of Agronomy, Mendel University of Agriculture and Forestry, Zemedelska 1, CZ-613 00 Brno, Czech Republic

**Keywords:** Metallothionein, Rats, Liver metabolism, Fungi, Vitamins, Mycotoxins, Brdicka reaction, Differential pulse voltammetry

## Abstract

Mycotoxins are secondary metabolites produced by microfungi that are capable of causing disease and death in humans and other animals. This work was aimed at investigation of influence of mouldy wheat contaminated by pathogenic fungi producing mycotoxins on metallothionein levels in hepatic tissue of rats. The rats were administrating feed mixtures with different contents of vitamins or naturally mouldy wheat for 28 days. It was found that the wheat contained deoxynivalenol (80 ± 5 μg per kg of mouldy wheat), zearalenone (56 ± 3 μg/kg), T2-toxin (20 ± 2 μg/kg) and aflatoxins as a sum of B1, B2, G1 and G2 (3.9 ± 0.2 μg/kg). Rats were fed diets containing 0, 33, 66 and 100% naturally moulded wheat. Control group 0, 33, 66 and 100% contained vitamins according to Nutrient Requirements of Rats (NRC). Other four groups (control group with vitamins, vit33, vit66 and vit100%) were fed on the same levels of mouldy wheat, also vitamins at levels 100% higher than the previous mixtures. We determined weight, feed conversion and performed dissection to observe pathological processes. Changes between control group and experimental groups exposed to influence of mouldy wheat and experimental groups supplemented by higher concentration of vitamins and mouldy wheat were not observed. Livers were sampled and did not demonstrate significant changes in morphology compared to control either. In the following experiments the levels of metallothionein as a marker of oxidative stress was determined. We observed a quite surprising trend in metallothionein levels in animals supplemented with increased concentration of vitamins. Its level enhanced with increasing content of mouldy wheat. It was possible to determine a statistically significant decline (p<0.05) between control group and groups of animals fed with 33, 66 and 100% mouldy wheat. It is likely that some mycotoxins presented in mouldy wheat are able to block the mechanism of metallothionein synthesis.

## 1. Introduction

### 1.1. Fungi producing mycotoxins

Approximately 150 species of fungi including moulds, yeasts and mushrooms, especially of the genera *Aspergillus*, *Penicillium* or *Fusarium*, can produce highly toxic substances called mycotoxins [1–5]. These highly toxic substances pose a threat for animal organisms due not only to their acute toxicity, but also the toxicity of their metabolites. However, the exact mechanisms of their metabolization are still unclear. Human as well as animal poisoning induced by mycotoxins are called mycotoxicoses. The most frequent effects of mycotoxins are mutagenic, carcinogenic, teratogenic, goitrogenic, nephrogenic and oestrogenic [6–11]. Levels of mycotoxins in cereals and fodders relate with the fact that cereals as well as fodders are very porous due to the structure of the grain surface, which simultaneously affects the access of oxygen [12,13]. Therefore, the whole surface (pericarp) of cereal grains could be highly contaminated by spores of pathogenic micromycetes already during maturation of cereal grains directly on plants. Number of spores or direct content of mycotoxins in surface layer of cereal grains depends on temporality of harvest. Mycotoxins also occur in stacks of straw, surface layers of silage and contaminated grain fodders. In addition, mycotoxins were also determined in decaying leaves of rape in winter and in early spring seasons [14–16].

### 1.2. The most important mycotoxins

Aflatoxins are toxic secondary metabolites produced by some fungi, especially of genus *Aspergillus* (above all by two species - *Aspergillus flavus* and *Aspergillus parasiticus*). These mycotoxins are toxic for homoeothermic animals including human and induce mycotoxicosis called aflatoxicosis. Aflatoxins called B1, B2, G1 and G2 are the most investigated, but more aflatoxins based on structural modifications of aforementioned ones have been determined. Aflatoxins were discovered at the beginning of the sixties of the past century due to an epidemic known as turkey X disease during which more than 100,000 young turkeys in the areas surrounding London died after feeding with grain fodders containing toxic peanut flour. Several modes of poisoning have been identified in animals, mainly due to consumption of mouldy wet fodder. When ruminants are fed with mouldy fodder, mycotoxins can pass into milk and poison young animals. Presence of aflatoxin is largely associated with commodities produced in the tropics and subtropics, such as groundnuts, other edible nuts, figs, spices and maize.

Besides poisoning by mycotoxins due to mouldy fodders and other foodstuffs, other important route for mycotoxin intake, especially of aflatoxins, is connected with professional hazards. Manufacturing of fodders and pasturages based on cereal grains and their manipulation presents a very significant problem, because these products are obviously contaminated by aflatoxins. These mycotoxins are able to enter by an oral route or by absorption through the skin. Under certain conditions, such as moisture or temperature, *Aspergillus flavus* and *Aspergillus parasiticus* are able to grow intensively and biosynthesize aflatoxins on almost all organic substrates (including all agricultural commodities and wastes). Aflatoxins may be acutely toxic, carcinogenic, mutagenic and teratogenic. These compounds are primarily metabolised in the livers of vertebrates [17,18]. In addition, new technologies and procedures, which would be acceptable for easy, rapid and safe removing of mycotoxins out of environment, are still being investigated. Technologies for isolation of aflatoxins with bentonite, and modified bentonite as well as chitosan have been tested [19–24].

**Figure 1.**
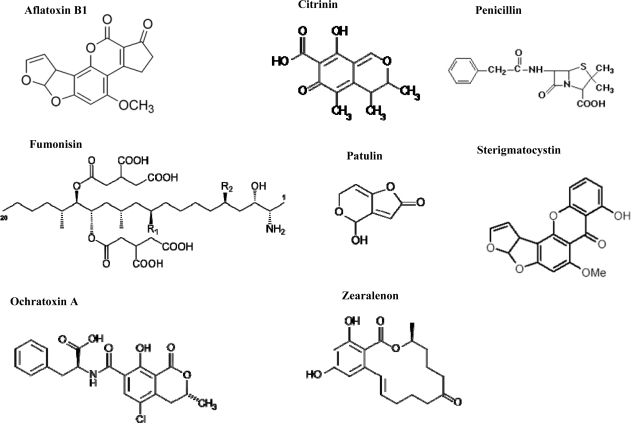
Chemical structures of mycotoxins produced by fungi of genera *Aspergillus*, *Penicillium* and *Fusarium*.

Citrinin is produced by some parasitic fungi of genera the *Penicillium* and *Aspergillus*. Citrinin usually occurs in cereals (or fodder based on them) and usually affects the kidneys and urinary bladder. Fumonisins are produced by some fungi of genus *Fusarium* (especially *F. moniliforme* and *F. proliferatum*). They cause several types of livestock disorders. Fumonisins can be found in cereals (especially in maize corns and products based on maize) and rice. Distinctive carcinogenic effects have been described, especially in the oesophagus. Penicillic acid is a compound chemically related to patulin. Its biological effects are similar to patulin (including cancerogenity). It often occurs in substrates stored under similar conditions suitable for fungi producing patulin. Ochratoxin A is a toxin produced by *Aspergillus ochraceus* and *Penicillium verrucosum* and is one of the most toxic and abundant food-contaminating mycotoxins. Ochratoxin A inhibits proteosynthesis, thus it can cause urinary tract tumours and is immunotoxic. This mycotoxin occurs in poorly stored food products, contaminated cereal grains and in other foodstuffs, such as coffee or fruit. Patulin is produced by many fungal species of the genera *Aspergillus*, *Byssochlamys* and *Penicillium*. Patulin is a secondary metabolite widespread in nature, especially because of its prevailing presence in rotting fruit, especially apples. Intoxications of cattle from rotting silage have been described with oedema as the main symptom. Patulin is not a particularly potent toxin, but a number of studies have shown that it is genotoxic, which has led to some theories that it may be a carcinogen. The mycotoxin sterigmatocystin is produced by a broader spectrum of fungal species of the genera *Aspergillus*, *Chaetomium* and *Emericella* and by some less known species of the genera *Bipolaris*, *Farrovia* and *Monocillium*. Sterigmatocystin affects the liver and is potentially carcinogenic. Tremorgens are a chemically heterogeneous group of mycotoxins that are produced by various species of microscopic and macroscopic fungi, above all of the genera *Aspergillus*, *Penicillium* (micromycetes) and *Claviceps* (macromycete). Tremorgens affect the nervous system, so symptoms of poisoning are tremors and convulsions. Trichothecenes, such as T-2 toxin, HT-2 toxin, deoxinivalenol or nivalenol, are very large family of chemically related sesquiterpenic mycotoxins produced by various species of *Fusarium*, *Myrothecium*, *Trichoderma*, *Trichothecium*, *Cephalosporium*, *Verticimonosporium* and *Stachybotrys*. Trichothecenes are toxic to animals and humans because they are able to inhibit protein, DNA, and RNA biosynthesis, damage cellular membranes and induce apoptosis in lymphatic and haematopoietic tissues. Their effects on health range from nausea and vomiting to retardation of growth, degeneration of immune, neural, and reproductive systems, and haemorrhagic illness. Zearalenone (ZON), also known as RAL and F-2 mycotoxin, is a potent estrogenic metabolite produced by some *Fusarium* species, which commonly infect cereal crops. Due to its estrogenic activity, zearalenone is known to disturb the ovulation cycle and reduce litter size in domestic animals, particularly in swine. In human, it has been hypothesized that foetal exposure to exogenous estrogenic substances may disturb male reproductive health, resulting in testicular cancer, cryptorchidism, hypospadias, and possibly reduced sperm quality [25–29]. Figure 1 shows chemical structures of certain mycotoxins. It is clear that mycotoxins are chemically very heterogeneous, but very often terpenic compounds [30]. Mycotoxins are very topical from the point of human and animal health protection view. Therefore it is not surprise that various scientific journals announce special issues devoted to these substances [21–24,30–57].

### 1.3. Biochemical effects of mycotoxins

**Figure 2.**
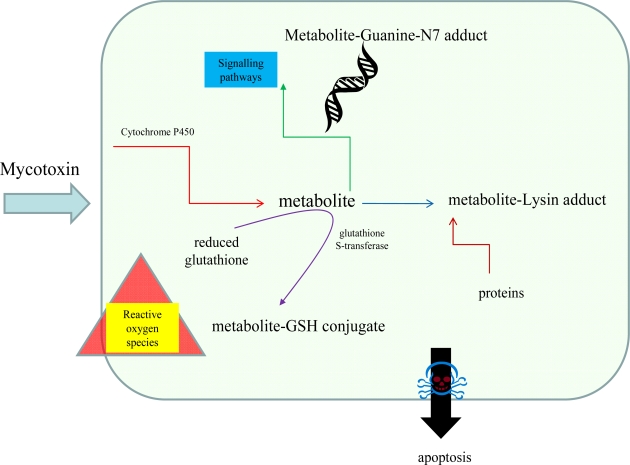
Simplified scheme of mycotoxin metabolism in vertebrates.

Interactions of mycotoxins with biomolecules have been investigated intensively. Fundamental metabolic pathways affected by mycotoxins are shown in Figure 2. Recently it was published that ochratoxin A increased expression of mRNA for glutathione S-transferase (GST) gene, which had activated protein-1 (AP 1) and NF-E2 related factor-2 (Nrf 2) [4,58]. Patulin activation of ERK1/2 signalling pathway, which correlates with PAT-mediated oxygen radicals, was observed [59]. Satratoxin H is thought to induce apoptosis of PC12 cells through the activation of p38 mitogen-activated protein kinase (MAPK) and c-Jun N-terminal kinase (JNK) in a glutathione (GSH)-sensitive manner [60]. Chemoprotective effects of flavonoid compounds against aflatoxins were investigated in hens. An experimental group of hens fed with fodder supplemented by doses of phenolic antioxidants demonstrated significant reductions of the toxic metabolites of the mycotoxins used [61]. In the event of rumen microbial ecosystem study, the effect of patulin was investigated. It was shown that the protective effect was caused by cysteine and reduced glutathione, however vitamin C and ferulic acid did not demonstrate an effect like this [62].

### 1.4. Metallothioneins and their relation to mycotoxins

Metallothioneins (MTs) are a group of low molecular mass (about 6.5 kDa) single-chain proteins. Four major isoforms (MT-1 through MT-4) have been identified in mammals [63,64]. They are found in cytoplasm, lysosomes, mitochondria and nuclei of cells. MT-1 and 2 have ubiquitous tissue distribution particularly in liver, pancreas, intestine, and kidney, whereas MT-3 is found in brain and MT-4 in skin [65]. Protection against metal toxicity is ensured mainly by MT-1 and MT-2, although MT-3 plays a role in Zn homeostasis in neurons [65,66]. Relation of MTs and mycotoxins are still unclear. There are only several works describing relation of glutathione to these molecules [67,68]. This work was aimed at investigation of the influence of mouldy wheat contaminated by pathogenic fungi producing mycotoxins on metallothionein levels in hepatic tissue of sewer rats. In addition, the effect of addition of vitamins to contaminated wheat was also studied.

### 2. Results and Discussions

It is well known that fungal spores producing mycotoxins are ubiquitous. Nevertheless, the presence of spores does not imply occurrence of toxic levels of mycotoxins in environment. Increased mycotoxin levels only occur under suitable conditions for mycelium growth (higher temperature and moisture). Temperature higher than 0 °C with optimum within the interval from 15 to 25 °C and moisture of foodstuffs higher than 16% are suitable for growth of microscopic fungi. However, changing of temperature and moisture does not always reduce metabolic and growth activity of fungi. It has been demonstrated that increased intake of mycotoxins can lead to a number of grave metabolic disorders in vertebrates. Metabolic changes were observed both in blood serum, where increase of hepatic enzymes and billirubin and reduction of total proteins were well evident, and in organs. Among organs liver, bile ducts, kidney, brain or testicles are most frequently mycotoxin-affected ones. It was demonstrated that mycotoxins interacted with intestinal mucous membrane and thus were able to permeate through this barrier. Experiments were carried out on intestinal mucous membranes of rats [69]. Concerning chronic administration of aflatoxin B-1 [AFB(1)] to rats enhanced number of hepatocellular and cholangiocellular carcinomas without affecting Kupffer and endothelial cells. The enzymatic conversion of AFB(1) to AFB(1)-8,9-epoxide is the critical step in the activation of the mycotoxin, while the conversion of AFB(1) to aflatoxin M(1) [AFM(1)], catalyzed by the AFB(1)-9a-hydroxylase, is considered to be a detoxification route for this toxin [70].

### 2.1. Effect of mouldy wheat on rats

**Figure 3.**
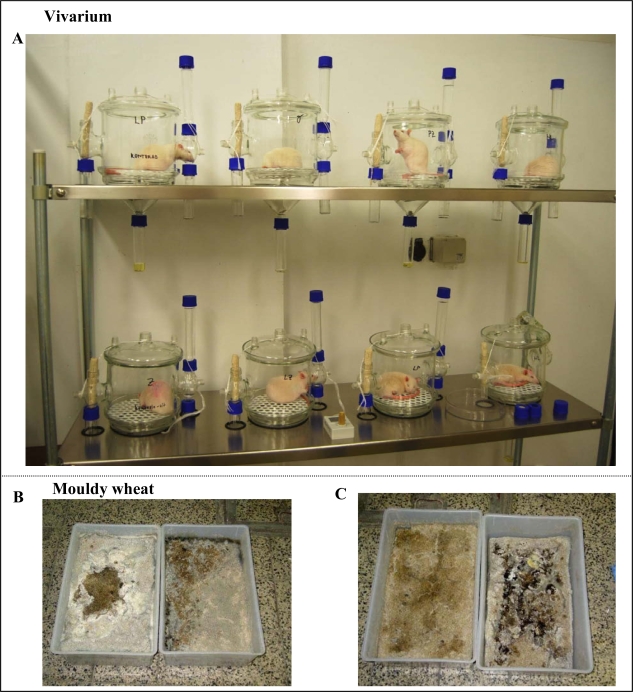
(A) Vivariums for rats with weight under 150 g. (B) Naturally mouldy wheat after fourteen or (C) twenty eight days of incubation.

As mentioned above, a number of experimental studies have been focused on investigation of mycotoxins’ influence on various experimental animals. In this study, we investigated the relationship between metallothionein levels and mycotoxin administration in rats (Figure 3A). For this purpose, experimental animals (28 days old; weight 65±5 g) were placed in vivariums and fed with mouldy wheat. Mouldy wheat after 14 and 28 days of the experiment is shown in Figures 3B, C. The mycotoxin content of the mouldy wheat was determined. It was found that the wheat contained deoxynivalenol (80 ± 5 μg per kg of mouldy wheat), zearalenone (56 ± 3 μg/kg), T2-toxin (20 ± 2 μg/kg) and aflatoxins as a sum of B1, B2, G1 and G2 (3.9 ± 0.2 μg/kg).

The experimental animals were fed with various mixtures containing different contents of mouldy wheat and vitamins *ad libitum* (Table 1). During the whole experiment, no changes in behaviour of the experimental animal groups were detected. The administered diet was designed based on common conventions and was composed of cereal grains, soya flour, starch, sunflower oil, minerals and vitamins, including addition of amino acid lysine. Rats were fed with diets containing 0, 33, 66 and 100% naturally moulded wheat. Control group 0, 33, 66 and 100% contained vitamins according to Nutrient Requirements of Rats (NRC). Other four groups (control group with vitamins, vit33, vit66 and vit100%) were fed on the same levels of mouldy wheat, but also vitamins at levels about 100% higher than the previous mixtures.

### 2.2. Changes in weight of rats

The highest increase of weight was observed in the third experimental week in the experimental group fed with mouldy wheat and in the experimental group with vitamin administration. In general, the increase in weight decreased with the quantity of mouldy wheat.

**Figure 4.**
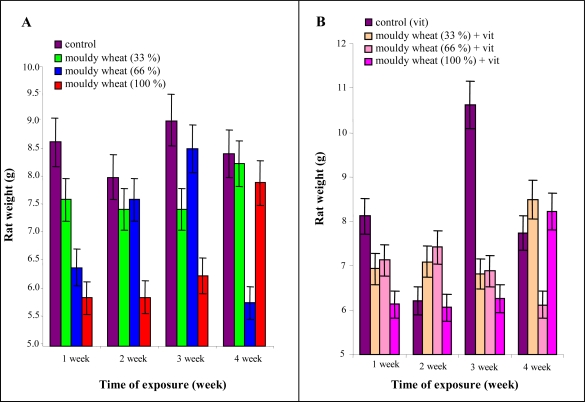
(A) Average weekly weight increase in rats administered various doses of mouldy wheat (Table 1) and vitamins according to NRC. (B) Average weekly weight increase in rats administered various doses of mouldy wheat (Table 1) and 100% higher dose of vitamins than suggested by NRC. Seven male rats per group.

Particularly, after 2 weeks the control, mouldy wheat 33 and 66% groups had the same increase in weight. After 3 weeks control and mouldy wheat 66% groups were alike. After 4 weeks mouldy wheat 66% group was less than the others (Figures 4A, B). The weight of the animals fed with 100% mouldy wheat differed significantly compared to control ones at the end of the experiment (Table 2). Other differences were not statistically significant.

### 2.3. Effect of mouldy wheat on feed conversion

**Figure 5.**
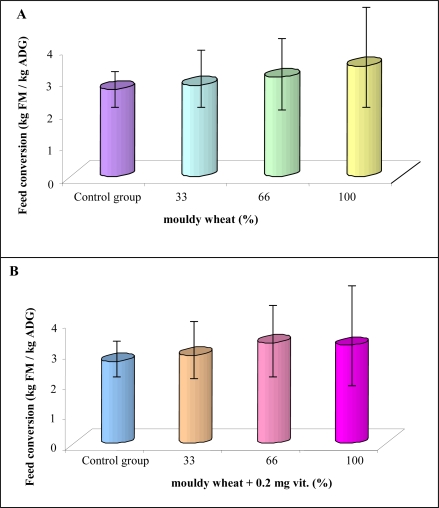
(A) Feed conversion of rats administered by various doses of mouldy wheat (Table 1) and vitamins according to NRC at the end of the experiment (4 weeks). We found out that the control group had the best feed conversion (2.70). The worst feed conversion had the group fed with 100% mouldy wheat (3.43). (B) Feed conversion of groups administered by various doses of mouldy wheat (Table 1) and 100% higher dose of vitamins than suggested by NRC at the end of the experiment (4 weeks). The control group had the best feed conversion (2.71). The worst feed conversion had the group with 66% mouldy wheat (3.32).

During the experiments, it was possible to monitor urine and excrements. Therefore, we were able to determine feed conversion. The conversion was slightly increased with increasing time of administration of mouldy wheat [from 3 to 3.5 kg FM per kg average daily gain (ADG); Figure 5A]. Concerning experimental groups supplemented by increased concentration of vitamins, a similar tendency was observed. Nevertheless 100% mouldy wheat administration caused slightly reduced feed conversion compared to 66% mouldy wheat administration (Figure 5B). Feed intake itself did not change much either in the mouldy wheat or in the mouldy wheat + vitamins experimental groups of rats. Particularly, control rats fed 4,101 g and 100% mouldy wheat rats 4,116 g. Nevertheless, the weight gain for these two groups was 1,519 g and 1,201 g, respectively, at the end of the experiment. Very similar phenomenon was observed in rats over-supplemented with vitamins. This phenomenon shows that mycotoxins have adverse effects on metabolism of rats as indicated by weight loss with the same feed intake compared to control specimens. Raymond *et al*. observed weight loss in horses administered grains naturally contaminated by mycotoxins [71]. There were no significant differences between feed conversion averages.

### 2.4. Dissection

**Figure 6.**
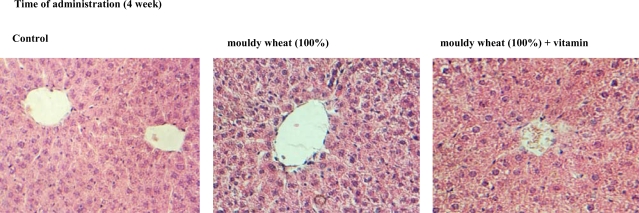
Typical histological changes in hepatic tissue structure in rats. The tissues were sampled after 4-week long exposition (magnification 120 ×).

At the very end of the experiment, animals were euthanized and subsequently dissection was carried out. The animals did not display any indicia of pathological processes. No changes between control group and experimental groups exposed to influence of mouldy wheat and experimental groups supplemented by higher concentration of vitamins and mouldy wheat were observed. Livers were sampled and did not demonstrate any significant changes in morphology compared to control. Histological and biochemical analysis of the sampled hepatic tissues were performed. Microphotos of hepatic tissues are shown in Figure 6. It clearly follows from the results obtained that there were no significant changes in structure between control and experimental groups from a histological point of view.

### 2.5. Effect of mycotoxins on metallothionein levels

In the following experiments levels of metallothionein as a marker of oxidative stress were determined. The oxidative stress obviously enhances levels of this protein in liver, kidneys and gonads [72–74]. The mentioned organs are characterized by higher metabolic activity.

**Figure 7.**
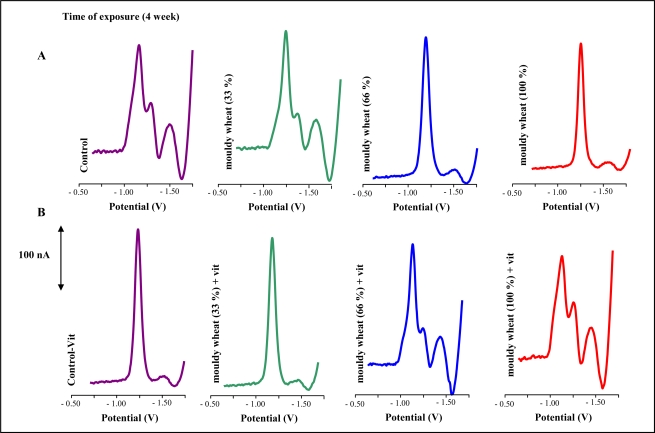
Typical DP voltammograms of the extracts obtained from livers of rats. The extracts were prepared according to protocol mentioned in the “Experimental” section; number of measurements = 5.

Electrochemical detection of metallothionein based on measurment of signals due to hydrogen evolution from solutions containing cobalt ions is very advantageous. These Brdicka signals of metallothionein are repeatable and sensitive to the presence of pg amounts of this protein [74–82]. Typical differential pulse voltammograms (DPV) of liver extracts are shown in Figures 7A,B. Catalytic signals called Cat2 proportional to MT concentration were well developed.

**Figure 8.**
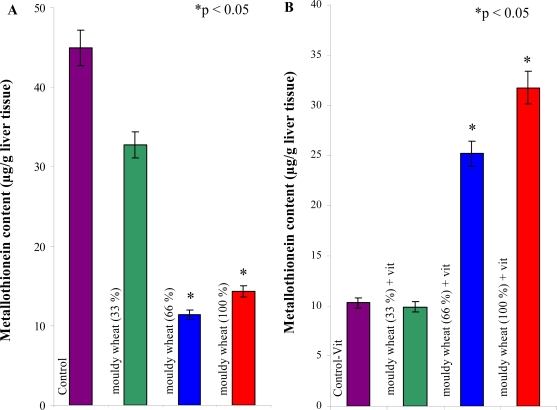
Changes of metallothionein levels in (A) rats exposed to mouldy wheat, (B) rats exposed to mouldy wheat and over-supplemented by vitamins at the end of the experiments (4 weeks); number of measurements = 5. Seven male rats per a group. Results were statistically treated by using of ANOVA, Scheffeho test. Differences with p < 0.05 were considered significant (vs. control only).

We observed a quite surprising trend in metallothionein levels in the animals supplemented with increased concentrations of vitamins (Figures 8A, B). The levels increased with increasing content of mouldy wheat. It was possible to determine statistically significant decline between control group and groups of animals fed by 33, 66 and 100% mouldy wheat (Table 3).

Moreover, we found a statistically significant difference between groups of animals fed by 100% mouldy wheat and groups of animals fed with 33 and 66% mouldy wheat. It follows from the results obtained that the hepatic metabolism of animals was negatively affected in spite of the fact that it was not possible to observe either recordable differences in weight increase and feed conversion or any histological results. Metabolism of hepatocytes is influenced due to mycotoxins administration. This fact is based on the changes in levels of metallothionein. In addition, levels of hepatic transaminases were slightly increased in a group exposed to mycotoxins, compared to the control group. Metallothionein responds very sensitively to increased metabolic demands of animals and most likely actively participates in organism protection against negative effects of mycotoxins. It is possible that metallothionein, as a transporter of zinc(II) ions, is intensively redistributed to tissue-specific transcriptional factors. It is likely that some mycotoxins presented in mouldy wheat are able to block the mechanism of metallothionein synthesis. This presumption can be supported by our experimental results. Besides metallothionein glutathione and detoxification enzyme glutathione-S-transferase also belong to key molecules participating in protection of an organism against xenobiotics [83]. Relation of this enzyme to mycotoxins levels has been investigated and protective effect of glutathione-S-transferase had been found [84].

In general, vitamins are important cofactors of many enzymes and thereby influence metabolic activity. The possibility to regulate levels of reactive oxygen species is one of their most important features. Mycotoxins increase oxidative stress reactions of organisms, as confirmed in the published literature [54,60,85–92]. This fact was demonstrated by increasing activities of the three most significant antioxidant enzymes (e.g. superoxide dismutase, glutathione peroxidase) in sheep exposed to sporidesmine [93], or on changes in glutathione levels [94–96]. If mice were supplemented with vitamins C and E together with selenium, protective effect against expected metabolic changes evoked by mycotoxins was observed [67]. There were also published results on the relationship between vitamin supplementation, particularly vitamin D [97–100], vitamin E [101–103] and vitamin B6 [104] and metallothionein levels.

**Figure 9.**
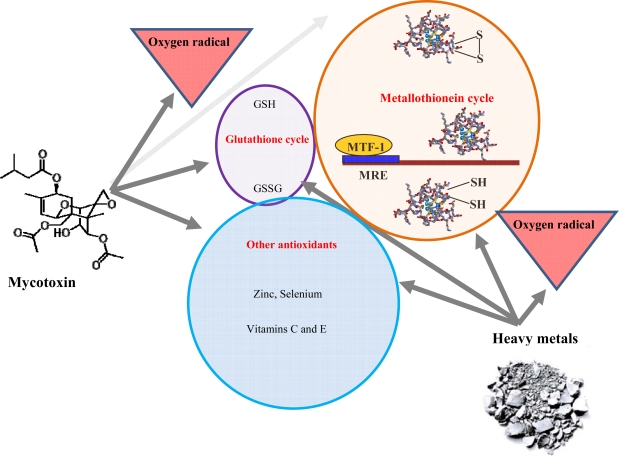
Simple scheme of mycotoxins affecting of MT synthesis. Mycotoxins produce oxygen radicals, which are scavenged by various mechanisms including MT synthesis.

We were interested in whether over-supplementation with vitamins can change the effect of mycotoxins on MT levels. Surprisingly, the level of metallothionein was enhanced with increasing doses of mycotoxins in rats fed these compounds and vitamins. Compared to non-vitamin groups we observed an adverse effect. The enhancement was significant for groups of animals fed with 66 and 100% mouldy wheat compared to control (Table 3). Moreover we observed statistical differences among experimental groups too. This fact can be related to interactions between vitamins and mycotoxins *in vivo*. These as yet unspecified interactions can alter the negative effects of mycotoxins on metallothionein synthesis. This assumption can be supported by the fact that level of metallothionein in control and control + vit groups significantly differed (p < 0.05, ANOVA, Scheffeho test). The effect of vitamins can be associated with the fact that these substances can decrease oxidative risk at animals of interest (Figure 9).

## 3. Experimental

### 3.1. Chemicals

Rabbit liver MT (MW 7143 g/mol), containing 5.9% Cd and 0.5% Zn, was purchased from Sigma Aldrich (St. Louis, USA). Co(NH_3_)_6_Cl_3_ and other chemicals used were purchased from Sigma Aldrich (Sigma-Aldrich, USA) unless noted otherwise. Stock standard solution of MT (10 μg mL^−1^) was prepared with A.C.S. water (Sigma-Aldrich, USA) and stored in the dark at −20 °C. Working standard solutions were prepared daily by dilution of the stock solutions with A.C.S. water. The pH value was measured using a WTW inoLab pH meter (Weilheim, Germany). All nutrients were purchased from Mikrop Čebín (Czech Republic).

### 3.2. Animals

Selected male *Wistar albino* laboratory rats of 28 days of age were used in our experiments. Experimental animals were kept in a vivarium with controlled air temperature (23 ± 1°C) and photo-period (12 hours day:12 hours night with maximal intensity 200 μE.m^−2^s^−1^). Rats were stabled in plastic cages with slotted floors. Tempered feed mixtures and drinking water were accessible *ad libitum.* Animals were weighed once a week and at the same time weight gain, feed intake, conversion and health state were monitored. Feed conversion was calculated according to the following equations: (feed intake)/(weight gain). The experimental animals were divided to eight groups (seven male rats per group). We used feed mixtures with different content of vitamins, naturally mouldy wheat or fungi (Table 1). The rats were administrating these mixtures for 28 days. Further, the animals were put to death and livers were sampled.

### 3.3. Fungi identification and quantification in mouldy wheat

Mouldy wheat (app. 20 g) was shaken in distilled water (180 mL) for 15 min. The suspension was diluted 10-fold with water. Then, ten times diluted suspension (1 mL) was introduced onto Petri dish with cultivation medium (Chloramphenicol Glucose Agar, Biokar Diagnostics, France). Fungi were cultivated for 125 hours at 25 °C. Detection of fungi species was performed microscopically. Total number of fungi was 2x10^6^ CFU/g (colony forming unit per a gram of mouldy wheat).

### 3.4. Preparation of biological samples

The animal tissue (app. 0.2 g) were transferred to a test tube and then deep-frozen by liquid nitrogen to disrupt cells. The frozen tissues were mixed with extraction buffer (100 mM potassium phosphate, pH 8.7) to a final volume of 1 mL and homogenised using an ULTRA-TURRAX T8 hand-operated homogenizer (IKA, Germany) placed in an ice bath for 3 min at 25,000 rpm [105]. The homogenate was centrifuged at 10,000 g for 15 min and at 4°C (Eppendorf 5402, USA). The processed tissues samples were prepared by heat treatment. Briefly, the sample was kept at 99 °C in a thermomixer (Eppendorf 5430, USA) for 15 min. with occasional stirring, and then cooled to 4 °C. The denatured homogenates were centrifuged at 4 °C, 15,000 g for 30 min. (Eppendorf 5402, USA). Heat treatment effectively denatures and removes high molecular weight proteins out from samples [77,106–109].

### 3.5. Electrochemical determination of metallothionein

Electrochemical measurements were performed with a 747 VA Stand instrument connected to 746 VA Trace Analyzer and 695 Autosampler (Metrohm, Switzerland), using a standard cell with three electrodes and cooled sample holder (4 °C). A hanging mercury drop electrode (HMDE) with a drop area of 0.4 mm^2^ was the working electrode. An Ag/AgCl/3M KCl electrode was the reference and glassy carbon electrode was auxiliary electrode. GPES 4.9 supplied by EcoChemie was employed. The Brdicka supporting electrolyte containing 1 mM Co(NH_3_)_6_Cl_3_ and 1 M ammonia buffer (NH_3_(*aq*) + NH_4_Cl, pH = 9.6) was used and changed per one analysis. DPV parameters were as follows: initial potential of −0.7 V, end potential of −1.75 V, modulation time 0.057 s, time interval 0.2 s, step potential 2 mV, modulation amplitude −250 mV, E_ads_ = 0 V. All experiments were carried out at a temperature of 4 °C (Julabo F12 cooler, Germany).

### 3.6. Statistical analyses

Data were processed using MICROSOFT EXCEL® (USA) and STATISTICA.CZ Version 8.0 (Czech Republic). Results are expressed as mean ± standard deviation (S.D.) unless noted otherwise (EXCEL®). Statistical significances of the differences between MT levels and rat weight were determined using STATISTICA.CZ. Differences with p < 0.05 were considered significant and were determined by using of one way ANOVA test (particularly Scheffeho test), which was applied for means comparison.

## 4. Conclusions

Metallothionein can play important role in the process of mycotoxins detoxification. Their exact role is still unclear, but this protein is probably connected with redistribution of significant ions to transcriptional factors and interactions with oxygen radicals that may be generated by mycotoxins. In addition, we also discuss that levels of vitamins can have crucial effect on cellular processes including protection against mycotoxins.

## Figures and Tables

**Table 1.** Composition of feed mixtures.

	**Mouldy wheat without vitamins**				**Mouldy wheat with vitamins**			
**Ingredient (w/w, %)**	**0%**	**33%**	**66%**	**100%**	**0%**	**33%**	**66%**	**100%**
**Wheat**	60	40.2	20.4	-	60	40.2	20.4	-
**Mouldy wheat**	-	19.8	39.6	60	-	19.8	39.6	60
**Soybean meal 47.5%**	12	12	12	12	12	12	12	12
**Starch**	22.34	22.34	22.34	22.34	22.14	22.14	22.14	22.14
**Lysine 78%**	0.46	0.46	0.46	0.46	0.46	0.46	0.46	0.46
**Minerals premix**	3	3	3	3	3	3	3	3
**Vitamins premix**	0.2	0.2	0.2	0.2	0.4	0.4	0.4	0.4
**SUM**	**100**	**100**	**100**	**100**	**100**	**100**	**100**	**100**

**Table 2.** Statistical treatment of rats weight at the end of the experiment by using one way ANOVA, Scheffeho test. Differences with p < 0.05 were considered significant, N.S.S. means any statistical significance.

	**control**	**MW (33%)**	**MW (66%)**	**MW (100%)**
control		n.s.s.	n.s.s.	n.s.s.
MW (33%)	n.s.s.		n.s.s.	n.s.s.
MW (66%)	n.s.s.	n.s.s.		n.s.s.
MW (100%)	n.s.s.	n.s.s.	n.s.s.	

	control (vit)	MW (33%) + vit	MW (66%) + vit	MW (100%) + vit
control (vit)		n.s.s.	n.s.s.	n.s.s.
MW (33%) + vit	n.s.s.		n.s.s.	n.s.s.
MW (66%) + vit	n.s.s.	n.s.s.		p < 0.05
MW (100%) + vit	P <0.05	n.s.s.	n.s.s.	

MW … Mouldy Wheat.

n.s.s. … No Statistical Significance.

**Table 3.** Statistical treatment of metallothionein content by using one way ANOVA, Scheffeho test. Differences with p < 0.05 were considered significant, N.S.S. means any statistical significance.

	**control**	**MW (33%)**	**MW (66%)**	**MW (100%)**
**control**		n.s.s.	p < 0.05	p < 0.05
**MW (33%)**	n.s.s.		p < 0.05	p < 0.05
**MW (66%)**	p < 0.05	p < 0.05		n.s.s.
**MW (100%)**	p < 0.05	p < 0.05	n.s.s.	

	control (vit)	MW (33%) + vit	MW (66%) + vit	MW (100%) + vit
**control (vit)**		n.s.s.	p < 0.05	p < 0.05
**MW (33%) + vit**	n.s.s.		p < 0.05	p < 0.05
**MW (66%) + vit**	p < 0.05	p < 0.05		p < 0.05
**MW (100%) + vit**	p < 0.05	p < 0.05	p < 0.05	

MW … Mouldy Wheat.

n.s.s. … No Statistical Significance.
